# Directed pterygomaxillary disjunction versus direct downfracture in Le Fort I osteotomy: Application of a fracture quality framework using bilateral ten-point cone-beam computed tomography in a retrospective cohort of 205 patients

**DOI:** 10.4317/medoral.1882

**Published:** 2026-02-22

**Authors:** Íñigo Aragón-Niño, José Luis del-Castillo-Pardo-de-Vera, José Luis Cebrián-Carretero, Clara López-Martinez, Blanca Tapia-Salinas, Chongyang Zheng, Carlos Navarro-Cuellar

**Affiliations:** 1 Doctoral School of Medical and Surgical Sciences. Faculty of Medicine, Complutense University of Madrid. Madrid, Spain; 2 IdiPAZ Translational Research Group in OMFS and Head and Neck Cancer. La Paz University Hospital. Madrid, Spain; 3 Oral and Maxillofacial Surgery Department. La Paz University Hospital. Madrid, Spain; 4 Anesthesiology Department. La Paz University Hospital. Madrid, Spain; 5 Oral and Maxillofacial; Head and Neck Oncology Department. Shanghai Ninth People's Hospital; Shanghai Jiao Tong University School of Medicine. Shanghai, People’s Republic of China; 6 Oral and Maxillofacial Surgery Department. Gregorio Marañón General University Hospital. Madrid, Spain

## Abstract

**Background:**

Control of the pterygomaxillary junction (PMJ) fracture is critical in Le Fort I osteotomy. We tested whether a directed PMJ disjunction (osteotome-assisted intermediate release) improves fracture quality, symmetry, and alignment versus direct downfracture.

**Material and Methods:**

Single-center retrospective comparative cohort (January 2019-April 2025). Two hundred five consecutive patients underwent Le Fort I: directed disjunction (n=127) or direct downfracture (n=78). Postoperative cone-beam computed tomography at day 90-110 was scored with a bilateral ten-point map. Primary outcomes were per-side quality (poor/good/excellent) and a patient-level overall quality status (poor/fair/good/very good/excellent). Inclusive and strict "clean-cut," symmetry, and alignment were predefined. Group comparisons used chi-square or Fisher's exact tests (two-sided =0.05).

**Results:**

Directed disjunction shifted side-level quality toward inferior, contained patterns: "excellent" 59.1% vs 6.4% (right) and 48.0% vs 12.8% (left); "poor" 22.0% vs 91.0% and 29.9% vs 85.9% (all p&lt;0.001). Patient-level status improved (poor 38.6% vs 98.7%; excellent 37.8% vs 1.3%; p&lt;0.001). Perfect symmetry rose to 49.6% vs 1.3% and correct alignment to 61.4% vs 1.3% (p&lt;0.001). Maxillary tuberosity involvement decreased from 60.3%/56.4% (right/left) without disjunction to 2.4%/5.5% with disjunction. Inclusive and strict clean-cut were higher with directed disjunction (69.3% vs 37.2% and 53.5% vs 9.6%; p&lt;0.001).

**Conclusions:**

Within a standardized early postoperative window, directed PMJ disjunction was associated with superior fracture quality, greater bilateral coordination, and fewer undesired trajectories than downfracture. Adoption of a targeted release and standardized reporting is supported. Interpretation is limited by the retrospective single-center design and focus on fracture behavior without complication or long-term outcome analysis.

## Introduction

Precise control of the pterygomaxillary junction (PMJ) fracture during Le Fort I osteotomy is pivotal to safe mobilization of the maxilla. Undesired trajectories-particularly superior propagation along the pterygoid plates or extension into the maxillary tuberosity-complicate segment manipulation and may increase morbidity (1 , 2). A structured, high-resolution characterization of the PMJ fracture is therefore clinically relevant to improve predictability and safety.

Despite the centrality of PMJ release to Le Fort I, the literature remains fragmented: Most reports treat the osteotomy as a single technical block, rarely isolating and analyzing the PMJ step. Heterogeneity in anatomical landmarks, inconsistent nomenclature for fracture patterns, and limited use of standardized cone-beam computed tomography (CBCT) protocols have hindered cross-study comparisons and the development of reproducible quality criteria (3 - 13). Crucially, prior work has seldom compared PMJ fracture patterns between alternative techniques (direct downfracture versus a targeted, osteotome-assisted release). This gap leaves unanswered whether a directed disjunction can systematically steer the fracture toward anatomically favorable paths.

To address this unmet need, we implemented a postoperative CBCT framework with bilateral ten-point mapping. It records inferior/superior involvement of the medial and lateral pterygoid plates and the status of the maxillary tuberosity on each side. This side-specific registration captures vertical distribution, mediolateral components, and inter-side symmetry/alignment with greater granularity than conventional descriptions. Building on this framework, we operationalized "fracture quality" with explicit, anatomy-based criteria and defined inclusive and strict "clean-cut" patterns to benchmark desirable trajectories.

We conducted a retrospective comparative analysis to determine whether a directed PMJ disjunction (intermediate osteotome-assisted release) yields superior fracture quality-characterized by inferior involvement of both plates, reduced superior propagation and tuberosity involvement-and improved bilateral alignment/symmetry, compared with direct downfracture. We hypothesized that targeted release channels the fracture vector inferiorly and homogenizes the pattern between sides, enhancing control, reproducibility, and anatomical respect of the PMJ fracture line.

## Material and Methods

**Figure 1:**
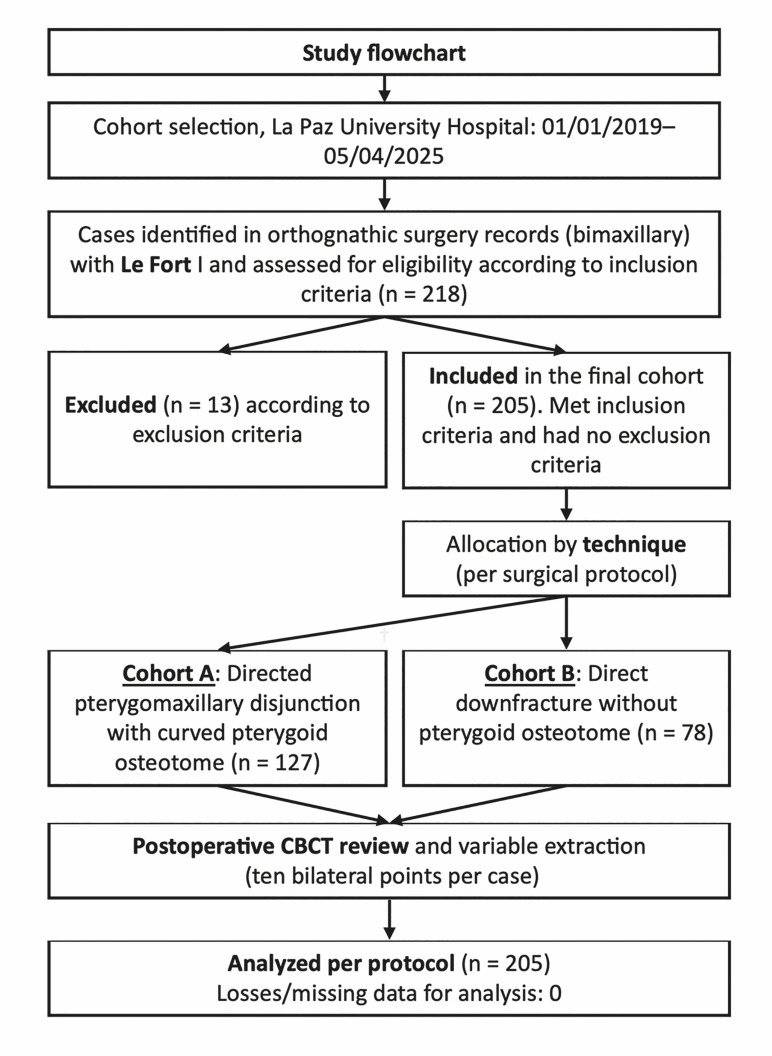
Study flowchart.

We conducted a single-center retrospective comparative cohort at the Department of Oral and Maxillofacial Surgery, Hospital Universitario La Paz, Madrid, Spain. The study period spanned from January 1, 2019 to April 5, 2025. From 218 initially screened interventions, 13 were excluded due to insufficient or non-verifiable clinical or radiological data, yielding a final cohort of 205 consecutive Le Fort I osteotomies (Figure 1).

1

All procedures were performed by the same surgical team under a uniform protocol. The decision to perform a directed pterygomaxillary disjunction was taken intraoperatively by the lead surgeon according to clinical judgment.

Participants: Eligibility and grouping

Eligible cases involved skeletally mature patients undergoing Le Fort I osteotomy for dentofacial deformity, with available postoperative cone-beam computed tomography (CBCT) and adequate short- to mid-term follow-up. Exclusions comprised craniofacial syndromes or congenital dysostoses affecting maxillary or pterygoid anatomy, prior maxillary or skull-base surgery, a history of midface trauma, combined or non-standard maxillary osteotomies, poor-quality postoperative CBCT that precluded reliable scoring, or inability to corroborate the PMJ technique from the operative record. Patients were classified retrospectively into directed disjunction or direct downfracture groups based on the documented intraoperative technique.

Interventions

All cases followed a standard Le Fort I approach under general anesthesia with an intraoral access. In the directed disjunction group, PMJ release was executed with a curved pterygoid osteotome (Obwegeser type) introduced between the maxillary tuberosity and the pterygoid plates, oriented medially and caudally approximately 30-45 degrees relative to the horizontal plane, and advanced with controlled taps to steer the fracture toward the inferior portions of both plates. In the direct downfracture group, maxillary separation was achieved without a specific pterygoid osteotomy.

Imaging protocol and reading workflow

Postoperative CBCT was obtained at approximately three months (acquisition window, postoperative day 90-110) using a homogeneous acquisition protocol at the same certified radiology center and device, with multiplanar reformatting in axial, coronal and sagittal planes and 3D reconstructions. Images were reviewed with dedicated DICOM software in a standardized workflow. Two maxillofacial surgeons independently and blindly assessed each scan; disagreements were resolved by consensus.

Fracture mapping framework and outcome definitions

**Figure 2:**
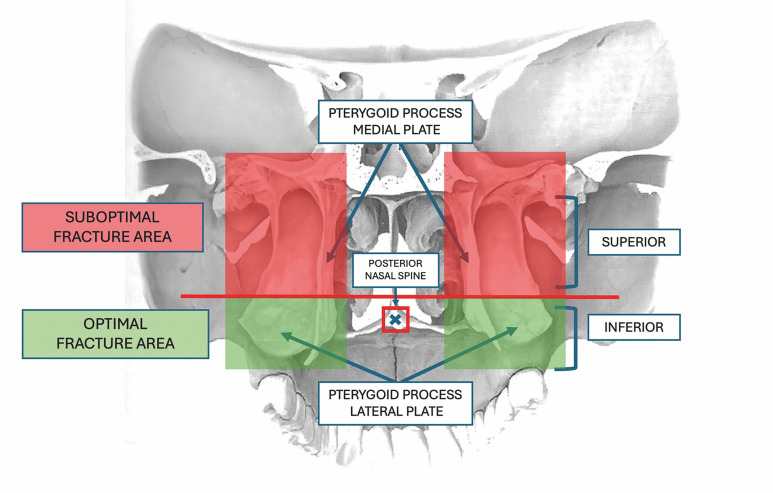
Anatomical schematic of the radiologic assessment of the fracture.

The primary endpoint was fracture quality at the pterygomaxillary junction, assessed on a side-specific map built from five anatomical checkpoints per side-the inferior and the superior portions of the medial and lateral pterygoid plates and the maxillary tuberosity. The inferior-superior split was defined by the axial plane through the posterior nasal spine; caudal to this plane was coded as inferior and cranial as superior (Figure 2).

2

Per-side quality had three ordered categories anchored in clinically desirable containment of the fracture line: Poor: Any superior plate portion or the tuberosity involved (regardless of inferior involvement). Good: Exactly one plate involved and confined to its inferior portion, without superior or tuberosity involvement. Excellent: Both plates involved but confined to their inferior portions, without superior or tuberosity involvement. To enable comparison with the "clean-cut" nomenclature used in prior literature, two pragmatic per-side surrogates were operationalized. An inclusive clean-cut required inferior involvement of both plates on a given side regardless of concurrent superior or tuberosity extension, whereas a strict clean-cut additionally required absence of any superior propagation and absence of tuberosity involvement on that side; reporting was performed both pooled by sides and stratified by right and left to avoid denominator ambiguity.

At the patient level, an ordinal bilateral index-overall quality status-integrated both sides by tallying the number of inferior plate portions involved across the four inferior segments provided no superior or tuberosity extension was present anywhere. Five ordered categories were defined: Poor if any superior or tuberosity extension existed on either side, fair if exactly one inferior portion was involved, good if two inferior portions were involved, very good if three inferior portions were involved, and excellent if all four inferior portions were involved; by construction, patterns confined to inferior portions are privileged and superior or tuberosity extensions are penalized.

Bilateral morphology was further summarized with symmetry and alignment outcomes. Symmetry was categorized as: Perfect symmetry; no symmetry with alignment; or no symmetry and no alignment. Alignment was also recorded as a binary variable using predefined bilateral criteria, allowing us to separate mirror-image concordance from directional concordance.

Statistical analysis

An anonymized database was assembled and exported to IBM® SPSS® Statistics (version 30.0.0, IBM Corp., Armonk, NY, USA) for analysis. Descriptive statistics summarized categorical variables as frequencies and percentages and age as mean with standard deviation. Normality was assessed with the Kolmogorov-Smirnov test. Between-group comparisons of categorical outcomes (including per-side clean-cut rates, patient-level overall quality categories, symmetry, and alignment) used the chi-square test or Fisher's exact test when expected counts were small. Age was compared between techniques with the Mann-Whitney U test given non-normality. All tests were two-sided with statistical significance set at P&lt;0.05.

## Results

Cohort

We analyzed 205 consecutive Le Fort I osteotomies, 127 with directed pterygomaxillary disjunction and 78 with direct downfracture. Women accounted for 71.2% and men for 28.8%. Age was comparable between techniques. Imaging acquisition and blinded readings were homogeneous across the series.

Per-side fracture quality (poor, good, excellent)

**Figure 3:**
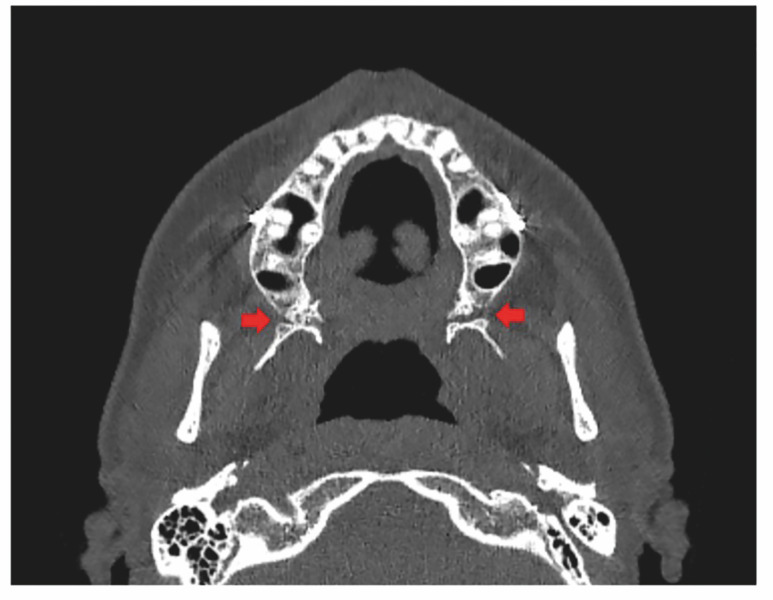
Fracture with inferior involvement, symmetric and aligned.

Directed disjunction shifted side-level quality decisively toward inferior, anatomically contained patterns on both sides, with significantly more "excellent" and fewer "poor" categories (all p&lt;0.001; Table 1). A representative case with inferior, symmetric, and aligned involvement is shown in Figure 3.

3

Bilateral overall quality status (patient-level index)

At the patient level, the bilateral ordinal index displaced toward higher grades with directed disjunction, indicating bilateral optimization under the quality framework (p&lt;0.001; Table 1).

Table 1

Symmetry and alignment

Bilateral morphology improved with directed disjunction, with a marked increase in perfect symmetry and correct alignment and the appearance of "no symmetry with alignment" only in the directed group (both p&lt;0.001; Table 2).

Table 2

Anatomic distribution of fracture involvement

Side-specific prevalence corroborated an inferior, controlled vector with directed disjunction: Inferior plate involvement predominated bilaterally, while superior portions and maxillary tuberosity involvement were markedly reduced compared with direct downfracture (Table 3).

Table 3

Undesired trajectories and clean-cut surrogates

Consistent with the above, the proportion classified as "overall quality: Poor" decreased and "excellent" increased with directed disjunction (p&lt;0.001; Table 1). Within the predefined surrogates, both inclusive and strict clean-cut were substantially more frequent with directed disjunction in pooled and side-specific analyses (p&lt;0.001; Table 4).

Table 4

**Figure 4:**
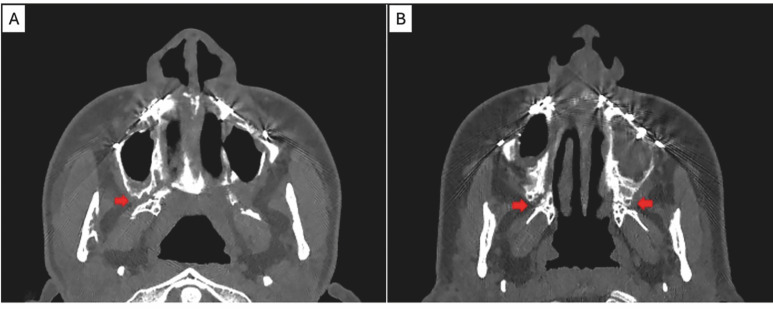
A: Fracture with involvement of the right maxillary tuberosity. B: Fracture with bilateral involvement of superior segments.

Representative examples of undesired trajectories are illustrated in Figure 4.

4

## Discussion

This study shows that introducing a directed release at the pterygomaxillary junction as an intermediate step in Le Fort I osteotomy improves the radiological quality of the fracture line, enhances bilateral coordination, and contains superior propagation. In a consecutive single-center cohort under a homogeneous acquisition and reading protocol, the directed approach shifted side-level categories away from poor patterns toward excellent, increased both inclusive and strict clean-cut rates, and displaced the patient-level overall quality status from the lowest grade toward intermediate and excellent grades. These improvements were accompanied by marked gains in bilateral symmetry and alignment, indicating a technique-dependent enhancement in control of the fracture trajectory.

A plausible mechanistic explanation is vector guidance imparted by the osteotome when introduced with a medial and caudal orientation. By steering separation toward the inferior portions of the medial and lateral pterygoid plates, the release converts a largely uncontrolled downfracture into a directed disjunction, which is reflected radiologically by inferior confinement and a sharp reduction in maxillary tuberosity involvement. The reproducible operative description used here-curved Obwegeser osteotome advanced at approximately 30-45 degrees relative to the horizontal plane with palatal counter-support-provides a technical substrate consistent with the observed bilateral containment.

Placed in context, heterogeneity in prior reports has stemmed from disparate taxonomies and incomplete isolation of the pterygomaxillary step within the Le Fort I sequence (14 - 17). Our bilateral ten-point cone-beam computed tomography framework directly addresses this gap by standardizing landmarks, capturing vertical distribution and mediolateral components on each side, and explicitly registering maxillary tuberosity status. The side-level ordinal quality scale (poor, good, excellent) and the patient-level overall quality status (poor, fair, good, very good, excellent) align radiological surrogates with clinically desirable endpoints and permit transparent comparison between techniques. When mapped onto the "clean-cut" nomenclature used in the literature, the present results demonstrate that directed disjunction increases both inclusive and strict clean-cut patterns, thereby reconciling differences in definitions while emphasizing the common goal of inferiorly oriented, anatomically respectful trajectories.

The methodological choices strengthen internal validity and reproducibility. A prespecified postoperative imaging window of 90-110 days was selected to minimize remodeling bias and approximate the original fracture path; readings were performed independently by two maxillofacial surgeons blinded to technique, with consensus resolution of discrepancies; and all examinations followed a uniform protocol at a single certified center. Together, these elements support the robustness of the quality framework and its applicability to routine audit.

Clinical implications are immediate. Incorporating a directed pterygomaxillary release can increase predictability during downfracture by producing symmetric, aligned fractures confined to the inferior plate portions, which facilitates segment manipulation and may reduce intraoperative instability. The quality framework itself is readily transferable to postoperative cone-beam computed tomography follow-up, enabling programmatic quality improvement, resident training, and inter-institutional benchmarking using shared definitions.

Limitations include the retrospective, single-center design and surgeon-driven selection of technique, which introduce potential selection and learning-curve effects despite the consecutive nature of the cohort and homogeneous protocols. The imaging window captures the early postoperative state rather than long-term remodeling; however, the chosen window is appropriate for assessing the primary construct of fracture quality. Finally, this article intentionally focuses on fracture behavior and does not analyze complications, operative time, or long-term occlusal and skeletal stability, which are addressed elsewhere.

Future studies should prospectively validate the framework across centers, test sensitivity to technical variables such as osteotome geometry and insertion angulation and compare directed disjunction with alternative instruments using the same quality definitions. Integrating biomechanical and cadaveric modeling could refine the mechanistic basis for vector guidance, and coupling radiological quality with patient-relevant outcomes and longer-term skeletal metrics will further establish clinical utility.

## Conclusions

In conclusion, a directed pterygomaxillary disjunction, assessed through a standardized bilateral ten-point cone-beam computed tomography framework, yields inferiorly contained and bilaterally coordinated fracture patterns in Le Fort I osteotomy. These findings support incorporating a targeted release step into routine practice and adopting standardized post-hoc quality reporting to improve comparability and drive iterative gains in safety and predictability.

## Figures and Tables

**Table 1:** Table Comparison of fracture quality outcomes by use of directed disjunction.

Variables	Categories	Directed disjunction				
		No (N=78)		Yes (N=127)		Pvalue
		N	(%)	N	(%)	
Right-side quality	Poor	71	-91	28	-22	<0,001
	Good	2	-2,6	24	-18,9	
	Excellent	5	-6,4	75	-59,1	
Left-side quality	Poor	67	-85,9	38	-29,9	<0,001
	Good	1	-1,3	28	-22	
	Excellent	10	-12,8	61	-48	
Overall quality	Poor	77	-98,7	49	-38,6	<0,001
	Fair	0	0	0	0	
	Good	0	0	15	-11,8	
	Very good	0	0	15	-11,8	
	Excellent	1	-1,3	48	-37,8	

1

**Table 2:** Table Comparison of symmetry and alignment by surgical technique.

Variables	Categories	Directed disjunction				
		No (N=78)		Yes (N=127)		Pvalue
		N	(%)	N	(%)	
Symmetry	No symmetry and no alignment	77	-98,7	49	-38,6	<0,001
	No symmetry with alignment	0	0	15	-11,8	
	Perfect symmetry	1	-1,3	63	-49,6	
Alignment	No	77	-98,7	49	-38,6	<0,001
	Yes	1	-1,3	78	-61,4	

2

**Table 3:** Table Prevalence of fractures by group (n and %).

Fracture sites(10 observations per patient)		Direct downfracture (N=78)	Directed disjunction (N=127)	Total (N=205)
Right side	Maxillary tuberosity	47 (60.3%)	3 (2.4%)	50 (24.4%)
	Medial pterygoid plate - superior portion	20 (25.6%)	8 (6.3%)	28 (13.7%)
	Medial pterygoid plate - inferior portion	34 (43.6%)	121 (95.3%)	155 (75.6%)
	Lateral pterygoid plate - superior portion	23 (29.5%)	21 (16.5%)	44 (21.5%)
	Lateral pterygoid plate - inferior portion	35 (44.9%)	99 (78.0%)	134 (65.4%)
Left side	Maxillary tuberosity	44 (56.4%)	7 (5.5%)	51 (24.9%)
	Medial pterygoid plate - superior portion	23 (29.5%)	14 (11.0%)	37 (18.0%)
	Medial pterygoid plate - inferior portion	40 (51.3%)	114 (89.8%)	154 (75.1%)
	Lateral pterygoid plate - superior portion	21 (26.9%)	24 (18.9%)	45 (22.0%)
	Lateral pterygoid plate - inferior portion	38 (48.7%)	83 (65.4%)	121 (59.0%)

Percentages are calculated within each technique group.

**Table 4:** Table Clean-cut equivalents by technique (inclusive and strict), cumulative frequencies and side-specific breakdown.

Technique	Definition	Granularity	Side	N/N	%
Directed disjunction	Inclusive clean-cut	Pooled (by sides)	-	176/254	69,3
	Strict clean-cut	Pooled (by sides)	-	136/254	53,5
		By side	Right	75/127	59,1
			Left	61/127	48,0
Direct downfracture	Inclusive clean-cut	Pooled (by sides)	-	58/156	37,2
	Strict clean-cut	Pooled (by sides)	-	15/156	9,6
		By side	Right	5/78	6,4
			Left	10/78	12,8

4

## Data Availability

Declared none.
